# Analysis of Clinical Features and Next-Generation Sequencing of 12 Tuberous Sclerosis Families in China

**DOI:** 10.3389/fmed.2022.840709

**Published:** 2022-05-27

**Authors:** Xu Wang, Wenda Wang, Yang Zhao, Zhan Wang, Yushi Zhang

**Affiliations:** Department of Urology, Peking Union Medical College Hospital, Chinese Academy of Medical Sciences and Peking Union Medical College, Beijing, China

**Keywords:** tuberous sclerosis complex, next-generation sequencing, clinical feature, gene mutation, family

## Abstract

**Background:**

Tuberous sclerosis complex (TSC) is a rare autosomal dominant genetic disease with systemic organ involvement. So far, only a few TSC families in China have been reported. Therefore, more data on the clinical and genetic features of TSC families are required.

**Materials and Methods:**

We retrospectively analyzed 12 TSC family probands and their family members. Next-generation sequencing (NGS) has been applied to confirm the type of TSC mutation along with a detailed physical examination.

**Results:**

In this study, twenty-seven patients in 12 TSC families were reported, including 12 male and 15 female patients, aged 8–67 years. Skin lesions were detected among all patients with TSC, including 25 cases of facial angiofibromas, 18 cases of hypomelanotic macules, 15 cases of ungual fibromas, and 13 cases of shagreen patch. Other clinical features were also revealed: 14 cases of renal angiomyolipoma, 6 cases of subependymal nodules (SENs), and 3 cases of lymphangioleiomyomatosis. All twenty-seven patients with TSC were tested by NGS. Totally, *TSC2* mutations were reported in 19 cases (7 frameshift mutations, 10 nonsense mutations, and 2 missense mutations), *TSC1* mutations were reported in 4 cases (4 nonsense mutations), and 4 cases were genetically negative. The novel causal mutations (*TSC2*: c.208dup, c.1874C > G, c.1852del) identified in three families were first reported in TSC.

**Conclusion:**

Our findings expand the mutation spectrum of patients with TSC in China. The clinical characteristics can vary among patients with TSC with the same pathogenic mutation. The genetic results and summary of clinical features of 12 TSC families contribute to a more accurate diagnosis and further genetic counseling.

## Introduction

Tuberous sclerosis complex (TSC) is an autosomal dominant genetic disease with an incidence rate of 1/6,000–10,000 (1). The pathogenesis of TSC is well established. The mutation of the *TSC1* or *TSC2* gene, which encodes hamartin and tuberin, directly results in excessive activation of the mammalian target of rapamycin (mTOR) pathway and leads to multiple disorders, such as cell growth, proliferation, and angiogenesis. TSC can affect almost every organ, such as kidney (2), skin (3), brain (4), heart (5), lung (6), and retina (7).

The clinical features of TSC are complicated. According to the second International Tuberous Sclerosis Complex Consensus Conference, 11 major features (facial angiofibromas, hypomelanotic macules, renal angiomyolipomas, cardiac rhabdomyomas, pulmonary lymphangioleiomyomatosis, etc.) and six minor features (“Confetti” skin lesions, dental enamel pits, multiple renal cysts, etc.) were confirmed as clinical diagnostic criteria. Besides, the presence of a pathogenic mutation in *TSC1* or *TSC2* was another independent diagnostic criterion regardless of the clinical findings (8).

To date, several cohorts have been reported based on TSC patients with TSC from different countries (9–11). As a rare genetic disease, systematic data on adult patients with TSC, especially with TSC families in China, are lacking. Here, our study reported clinical features, as well as genetic mutations by next-generation sequencing (NGS) to analyze the characteristics of TSC families in China and expanded a novel spectrum of criteria.

## Materials and Methods

### Participants

Medical records of TSC family patients registered at Peking Union Medical College Hospital from January 2010 to January 2020 were reviewed. At the 2012 International Tuberous Sclerosis Complex Consensus Conference, a definite diagnosis was defined as including 2 major criteria or 1 major criterion and ≥2 minor criteria, as well as pathological genetic mutations, whereas suspected diagnosis was defined as including 1 major criterion or ≥2 minor criteria. Overall, 197 patients enrolled in our hospital's TSC cohort were retrospectively analyzed, and 12 TSC families were screened out. In addition to the proband, we conducted complete TSC-related examinations (such as physical examination, imaging examination, and NGS examination) among the immediate relatives of the proband and suspected patients with TSC in the family. Written informed consent was obtained from the individuals and minors' legal guardians for the publication of any potentially identifiable images or data included in this article.

### Next-Generation Sequencing

Written informed consent was obtained from all the participants or their guardians when peripheral blood was collected. This study was approved by the ethics committee of Peking Union Medical College Hospital. The whole exomes were detected by NGS. Peripheral blood (5 ml) from all the probands and confirmed/suspected relatives was extracted. Genomic DNA was extracted from peripheral blood leukocytes *via* the QIAamp DNA Blood Mini Kit (Qiagen, Hilden, Germany), guided by the recommended protocol. The DNA library was prepared in accordance with standard operating procedures. Briefly, genomic DNA was fragmented into 200–250 bp fragments using a Covaris LE220(BGI—Shenzhen, China) sonicator, and then the fragments were purified with an Agencourt AMPure XP kit (BGI - Shenzhen, China). The 3′ and 5′ ends of the purified DNA fragments were modified by T4 DNA polymerase and dNTP, and terminal A was added by incubation with dATP and Klenow 3'-5' exoenzymes according to the Illumina standard protocol. Ligation-mediated polymerase chain reaction (PCR) and purification were performed. Then, purified DNA fragments were hybridized with customized gene fragment capturing chips (Roche NimbleGen, Madison, WI). The qualified DNA samples were amplified with the high-fidelity DNA polymerase and sequenced with the Illumina HiSeq 2500 sequential platform (Illumina, San Diego, CA) for bidirectional sequencing of 90 cycles. The original image data were processed with Illumina base-calling software (V.1.7, Illumina). Sequentially alignment was performed on qualified original reads using Burrows-Wheeler Aligner software (BGI-Shenzhen, Shenzhen, China). The bam data were used for target reads coverage, sequencing depth computation, single nucleotide polymorphism (SNP) calling, insertion-deletion annotation, and copy number variation detection. The average sequencing depth of the target region was 2000X, and 98% of the target sequences were more than 100X. First, SOAPsnp software (BGI-Shenzhen, Shenzhen, China) and Samtools pileup software (BGI-Shenzhen, Shenzhen, China) are used to call SNP and insert-deletion respectively. Then, if the frequency of an SNP is >0.05 in 1,000 Genomes Project, HapMap, dbSNP, or BGI Local Database databases, it would be considered a polymorphism, not a pathogenic mutation. Finally, novel mutations were identified from the Leiden Open Variation Database (LOVD) and ClinVar database (https://www.ncbi.nlm.nih.gov/clinvar).

## Results

### Study Population and Basic Characteristics

**Figure 1:**
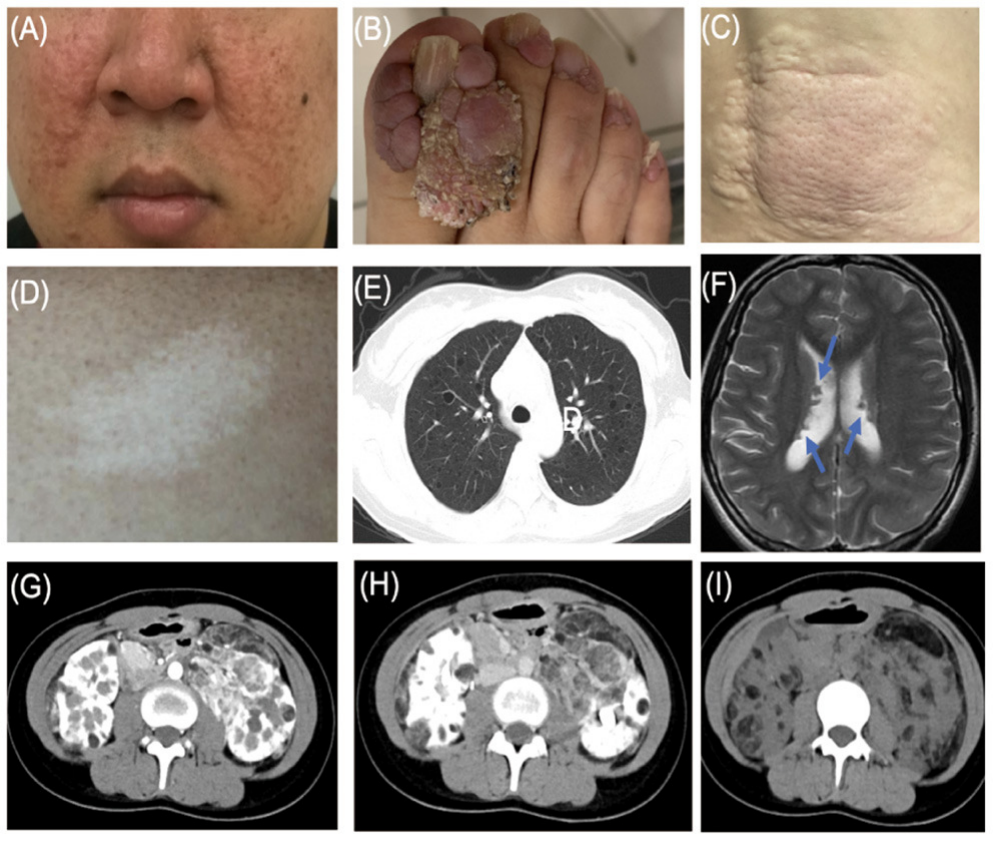
Typical features of tuberous sclerosis complex (TSC). **(A)** Facial angiofibromas: small rashes usually found on the nose and cheeks **(B)** Ungual fibromas: fibrous around the finger or toenails. **(C)** Shagreen patch: thickened raised skin usually found on the lower back. **(D)** Hypomelanotic macules. **(E)** LAM: Lymphangioleiomyomatosis. **(F)** Subependymal nodule (arrow). **(G)** Angiomyolipoma (arterial phase). **(H)** Angiomyolipoma (venous phase). **(I)** Angiomyolipoma.

**Figure 2:**
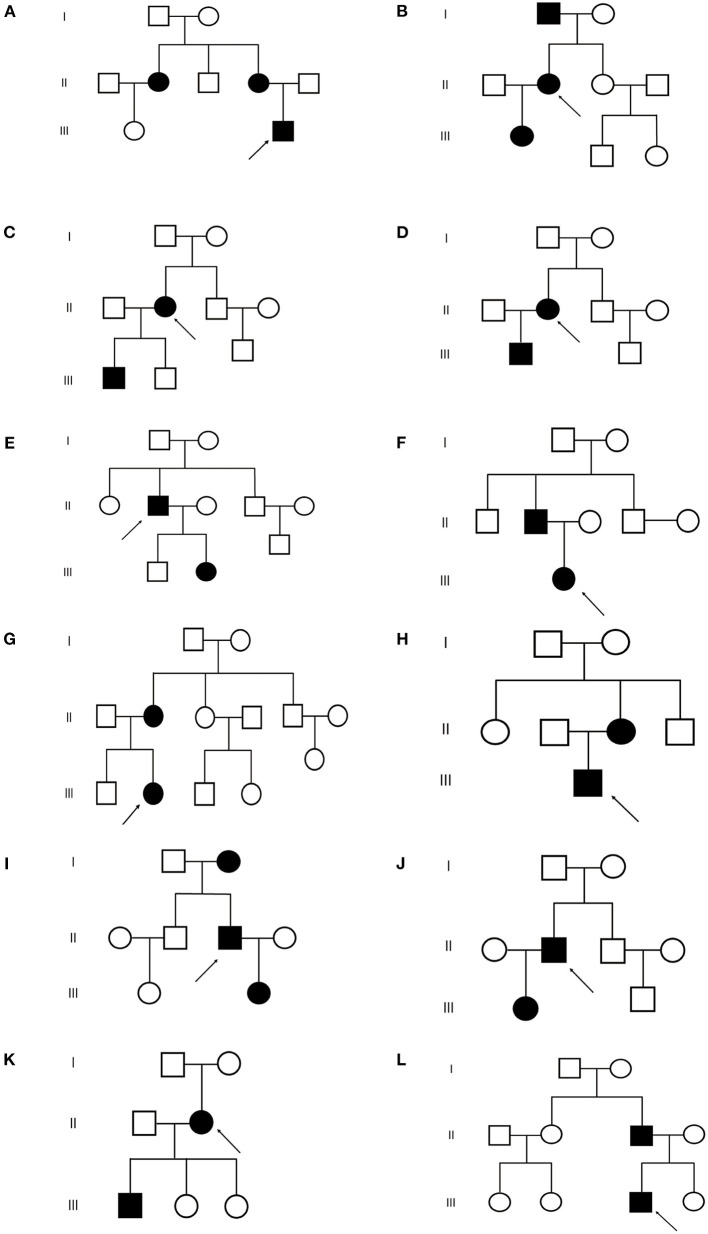
Pedigrees of the 12 TSC families. **(A–L)** Represent **(A–L)** family respectively. 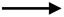: proband 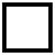: normal male 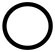: normal female 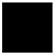: TSC male 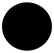: TSC female.

Among the 12 families included in the study, there were 27 patients, including 12 men and 15 women, with a male-to-female ratio of 1:1.25, aged 8–67 years old. The cause of admission among 12 probands was abdominal/waist discomfort (5 cases), skin lesions (4 cases), and renal mass by physical examination (3 cases). All patients had cutaneous manifestations: facial angiofibroma (25 cases, 92.6%) and hypomelanotic macules (18 cases, 66.7%) were the most common, followed by ungual fibromas (15 cases) and shagreen patch (13 cases). There were 14 cases of renal angiomyolipoma, 6 cases of subependymal nodules (SENs), and 3 cases of pulmonary lymphangiomyomatosis. Three cases had a history of epileptic seizures. Two cases had hepatic angiomyolipoma, and polycystic changes in both kidneys were found in only 1 case. The clinical manifestations of the proband and family members are shown in Figure 1 and Table 1, and the pedigree is shown in Figure 2. Three families had more than two patients with TSC. The detailed histories of these three families were given below.

**Table 1:** Clinical features and pathogenic mutations of TSC families.

**Pathogenic mutation**					**Family**		**Gender**	**Age**	**Clinical features**								
**Gene**	**Nucleotide change**	**Protein change**	**Type**	**Diagnosis**					**FA**	**HM**	**SP**	**UF**	**AML**	**SEN**	**LAM**	**MRC**	**NH**
TSC2	c.208dup	p.Thr70Asnfs ^*^5	Frameshift	Definite	A	1^*^	M	21	✓	✓	✓		✓				✓
						2	F	45	✓	✓	✓	✓	✓				
						3	F	51	✓		✓	✓	✓				
	c.1047dup	p.Arg350^*^	Nonsense	Definite	B	1^*^	F	40	✓	✓					✓		
						2	F	16	✓		✓			✓			
						3	M	67	✓	✓		✓					
	c.4544_4547del	p.Asn1515 Serfs^*^60	Frameshift	Definite	D	1^*^	F	30	✓	✓		✓					
						2	M	8	✓		✓			✓			
	c.1874C>G	p.Ser625^*^	Nonsense	Definite	E	1^*^	M	45	✓	✓		✓	✓				✓
						2	F	22	✓	✓		✓	✓				
	c.3581G>A	p.Trp1194^*^	Nonsense	Definite	F	1^*^	F	12	✓		✓		✓		✓		
						2	M	38	✓	✓							
	c.1852del	p.Leu618 Cysfs^*^80	Frameshift	Definite	G	1^*^	F	30		✓		✓			✓	✓	
						2	F	57	✓	✓		✓					
	c.1831C>T	p.Arg611Trp	Missense	Definite	H	1^*^	M	18	✓		✓		✓				
						2	F	40	✓	✓		✓	✓				
	c.3685C>T	p.Gln1229^*^	Nonsense	Definite	I	1^*^	M	35		✓	✓	✓		✓			
						2	F	10	✓	✓		✓					
						3	F	64	✓		✓	✓					
					TSC2 Total	19 (73.4%)			17 (63.0%)	13 (48.1%)	9 (33.3%)	12 (44.4%)	8 (29.6%)	3 (11.1%)	3 (11.1%)	1 (3.7%)	2 (7.4%)
TSC1	c.2227C>T	p.Gln743Ter	Nonsense	Definite	C	1^*^	F	53	✓	✓	✓	✓	✓				
						2	M	25	✓		✓		✓	✓			
	c.733C>T	p.Arg245Ter	Nonsense	Definite	J	1^*^	M	58	✓		✓		✓				
						2	F	30	✓		✓		✓				
					TSC1 Total	4 (14.8%)			4 (14.8%)	1 (3.7%)	4 (14.8%)	1 (3.7%)	4 (14.8%)	1 (3.7%)	—	—	—
—	—	—	—	—	K	1^*^	F	38	✓	✓		✓					
						2	M	13	✓	✓		✓		✓			
—	—	—	—	—	L	1^*^	M	18	✓	✓			✓	✓			
						2	M	37	✓	✓			✓				
					No mutation Total	4 (14.8%)			4 (14.8%)	4 (14.8%)	—	2 (7.4%)	2 (7.4%)	2 (7.4%)	—	—	—
					Total	27			25 (92.6%)	18 (66.7%)	13 (48.1%)	15 (55.6%)	14 (51.9%)	6 (22.2%)	3 (11.1%)	1 (3.7%)	2 (7.4%)

* *Proband; M, male; F, Female; FA, facial angiofibromas; HM, hypomelanotic macules; SP, shagreen patch; UF, ungual fibromas; AML, angiomyolipoma; SEN, subependymal nodule; LAM, lymphangioleiomyomatosis; MRC, multiple renal cysts; NH, nonrenal hamartomas*.

#### Family A

The proband was a 21-year-old man. The patient underwent abdominal ultrasound during the admission physical examination, indicating multiple renal masses and uneven echo. The patient came to our outpatient department for further diagnosis and treatment. The patient had multiple facial angiofibromas on his face. Several hypomelanotic macules and shagreen patches were found on his lower back. Abdominal CT showed multiple angiomyolipomas in the liver and bilateral kidneys. As a result, the proband was diagnosed with TSC. Tracing the proband's family, the proband's mother and aunt also had TSC clinical characteristics (mother: facial angiofibromas, hypomelanotic macules, shagreen patches, ungual fibromas, and angiomyolipoma; aunt: facial angiofibromas, shagreen patches, ungual fibromas, and angiomyolipoma). An NGS examination was performed on the proband, his immediate relatives, and suspected patients with TSC in the family. The pathogenic *TSC2* mutation (NM_000548.4:c.208dup) was found in the proband, his mother, and his aunt only. No causal variants were screened out of the remaining ones.

#### Family B

The proband was a 40-year-old woman. The patient was admitted to the local clinic due to sudden right chest pain, chest tightness, and breathlessness during exercise. A CT scan of the chest revealed a right pneumothorax and multiple bullae in both lungs. After conservative treatment, the patient was admitted to the respiratory department of our hospital. The patient had facial angiofibromas and hypomelanotic macules on the back. Based on the physical and imaging examination, the proband was diagnosed with TSC. Tracing the proband's family, TSC was also diagnosed in the proband's daughter and father (daughter: facial angiofibromas, shagreen patches, and SEN; mother: facial angiofibromas, hypomelanotic macules, and ungual fibroma). After the examination of NGS on Family B, pathogenic *TSC2* mutation (NM_000548.4:c.1047dup) was found in the proband and her daughter and father.

#### Family I

The proband was a 35-year-old man. He was admitted to the emergency room of our hospital due to a fall injury. Head CT indicated that there was no intracranial hemorrhage but an SEN could be found. The patient went to the neurology department. Physical examination found hypomelanotic macules, shagreen patches, and ungual fibroma. TSC was also diagnosed in the proband's daughter and mother (daughter: facial angiofibromas, hypomelanotic macules, and ungual fibroma; mother: hypomelanotic macules, shagreen patches, and ungual fibroma). Through NGS, the pathogenic *TSC2* mutation (NM_000548.4:c.3685C>T) was found in the proband and her daughter and mother.

### Genetic Screening

**Figure 3:**
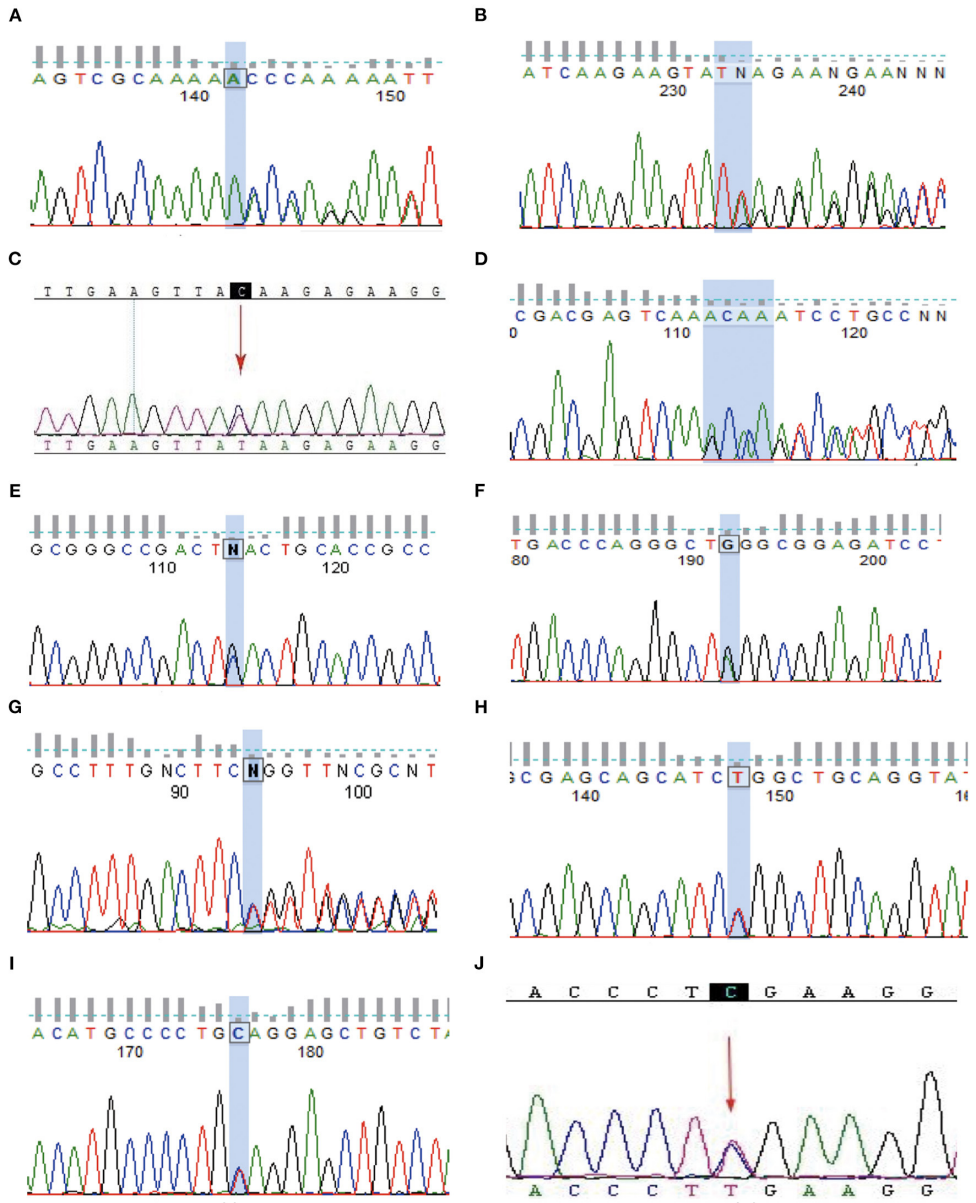
Sanger validation. **(A–J)** Represents genetic mutation in the **(A–J)** family, respectively.

In total, 8 families with *TSC2* mutations were identified. Families A, B, D, E, F, G, H, and I were affected by *TSC2* mutations. Family C and J were *TSC1*-mutated. No mutation was identified in the remaining 2 families. Remarkably, a total of 3 novel mutations were reported for the first time (Family A, E, and G). Frameshift mutations were identified in Family A, D, and G. Nonsense mutations were reported in Family B, C, E, F, I, and J, and missense mutation was detected in Family H (Table 1). The Sanger sequencing was used to validate the NGS results among TSC family members (Figure 3).

## Discussion

Tuberous sclerosis complex is an autosomal dominant genetic disease, where affected individuals have a more than 50% chance of transmitting the mutations. The outstanding feature of TSC is the diversity of clinical manifestations. In our study, we also found that patients with TSC who shared the same mutations in TSC families could vary in phenotype. In previous studies, Salussolia et al. also reported that even in the same family, the symptoms can vary significantly (12). According to clinical experience, most patients did not know about TSC before the onset of the disease, and some medical staff in primary hospitals also lacked relative knowledge, making missed diagnoses more common. Therefore, understanding the pathogenesis and common clinical symptoms will be able to contribute to increasing clinicians' awareness of TSC. The results of this study show that the most common characteristics of TSC families are facial angiofibroma and hypomelanotic macules. Therefore, we believe that the introduction of TSC skin characteristics should be emphasized in the TSC education work. Studies have shown that in patients with gene mutations, *TSC2* mutations account for more than 75–80%, which is much higher than *TSC1* (13). In this study, the ratio of *TSC2*/*TSC1* was 4.75:1, which conformed to the characteristics of *TSC2* mutation advantage. In the study by Cai et al. (14) and Dabora et al. (15), through genetic testing of patients with TSC, researchers found that compared with non-*TSC2* patients, patients with *TSC2* had a certain degree of connection with severe clinical phenotype. They had a higher frequency of seizures, more brain disorders (SEN and cortical tubers), more multiple kidney masses, and more severe skin lesions (facial angiofibromas and hypomelanotic macules). In our study, patients did not show significant differences in symptom severity. Additionally, we analyzed genotypic and phenotypic correlation in the same family, but unfortunately, we did not find a clear correlation. Due to the complexity of clinical manifestations of TSC and the “second hit” mechanism, even patients with the same mutation can still have a large difference in clinical characteristics.

Due to the multi-organ involvement of TSC, the clinical and imaging manifestations of patients are often complicated and diverse. Several results showed that skin and neurological manifestations were the most common in patients with TSC. Dermatological manifestations mainly include four of eleven major features (facial angiofibromas/fibrous cephalic plaques, hypomelanotic macules, shagreen patches, and ungual fibromas). Cutaneous manifestations were present in about 90% of patients with TSC, with facial angiofibroma and hypomelanotic macules account for 80–90% of the total (16). In our study, all the patients had at least one skin lesion. Facial angiofibroma (25/27, 92.6%) and hypomelanotic macules (18/27, 66.7%) claimed the top 2 cutaneous affection, which was consistent with the previous results. Patients with TSC are also at high risk for neurodevelopmental disorders, such as brain structural abnormality (SENs, cortical dysplasia, and subependymal giant cell astrocytoma) and neuropsychiatric disorders, described as refractory epilepsy and infantile seizures (17). In our study, only 6 cases had SENs. The 3 cases had a history of epileptic seizures. The 3 patients with a history of epilepsy in this study all experienced seizure onset in the first 2 years of life. In the TuberOus SClerosis registry to increase disease Awareness (TOSCA) involving 2,093 patients with TSC from 171 centers in 31 countries, 1,199 cases (57.3%) had facial angiofibroma and 1,748 cases (83.5%) had a history of seizures (18). The proportions of skin lesions and neuropsychiatric manifestations in our study were significantly different from those in TOSCA. On the one hand, the difference in proportion may be caused by the different sample volumes. On the other hand, some studies have proved that facial angiofibroma can progress with age and epilepsy usually starts in young children within 1 year of age (1, 19). Therefore, we thought that the influence of age composition on TSC disease progression is responsible for the above differences in results, considering that 63.3% of patients in the TOSCA study were <18 years old.

The kidney is one of the most vulnerable target organs of TSC. In addition to renal angiomyolipoma, the kidney can also be manifested as renal cysts, and may even be malignant (20). As a variant of angiomyolipoma, the renal epithelioid angiomyolipoma (EAML) has the characteristics of the predominance of the perivascular epithelial cell (PEC) and a unique morphologic and immunohistochemical panel (21). As a kind of rare tumor, EAML is statistically found in <1 in 10,000 of the population, accounting for 8% of surgically treated AML (22). Although the occurrence and development mechanism of EAML is still unclear, accumulating evidence certainly shows that EAML is malignant, characterized by strong aggressiveness and rapid progression (21). Compared with sporadic AML, which is mostly unilateral and single, tuberous sclerosis-associated renal angiomyolipoma (TSC-AML), described as bilateral and multiple, can be seen in 70–80% of patients with TSC (23). The size and number of AML will gradually increase with age. Compared with younger patients, the AML growth rate and incidence were higher in older adult patients (24). In our groups of TSC families, 13 cases had TSC-AML family history and only 1 case was sporadic, indicating the incidence of AML in TSC families has a certain tendency of family aggregation. Bilateral and multiple AMLs were scanned in all 14 patients by abdominal CT. Studies have clarified that the presence of end-stage renal disease, renal tumor rupture, and hemorrhage are the main causes of death in adult patients with severe TSC-AML. Therefore, for TSC patients with AML, abdominal imaging (e.g., CT and magnetic resonance imaging [MRI]) should be performed every 1–3 years throughout a patient's lifetime to follow renal lesions. Blood pressure as well as eGFR/ serum creatinine levels should be monitored to clarify the renal function and the burden of masses (25, 26). Based on the pathogenic mechanism of TSC, everolimus, known as an mTOR inhibitor, has been used as the first-line therapy for TSC (27). For refractory AML or AML at risk of malignancy, surgical treatment has become a new option. It should be noted that surgery should preserve renal function as much as possible and prolong life expectancy. However, given the high incidence of postoperative complications and the increased risk of renal insufficiency and end-stage renal failure in the future, further clinical studies are needed to confirm whether patients will benefit in the long term after surgery (25).

As a pulmonary disease, LAM is an important cause of pneumothorax and respiratory failure in patients with TSC. All 3 patients with LAM were women, which was consistent with our clinical experience and TSC cohort. It is currently believed that hormones play an important role in the development of LAM. Clinical observation shows that LAM almost occurs in women. LAM can progress during the period, pregnancy, and exposure to estrogenic medicine. The condition of LAM was relatively stable in postmenopausal women. In addition, studies have shown that LAM cells express estrogen and progesterone receptors (28). Lu et al. reported that estrogen increases the growth of TSC2-deficient cells by activating pyruvate kinase M2 (29). Therefore, female hormone levels may be the key factor causing the predominance of LAM in women. Besides, more research is needed for further understanding of LAM.

In our study, we sequenced 27 patients with TSC in 12 families and found that 19 cases in 8 families had *TSC2* gene mutations, described as 7 frameshift mutations and 10 nonsense mutations, and 2 missense mutations, compared with 4 *TSC1* cases and 4 cases without mutations. It is worth mentioning that the mutations in 3 families (Family A, E, and G) are the first reported, which further expands the TSC mutation map in Chinese patients. The mutation c.3685C>T from Family I was reported by Dabora et al. (15) and Rosset et al. (30). The nonsense mutation may probably lead to the early termination of the protein encoded. As early as 2005, c.4544_4547del of Family D was reported in Indians by Ali et al. (31). The loss of 4 bases in the coding region of the *TSC2* is speculated to cause a frameshift mutation, leading to premature termination of protein translation. According to the current evidence, the variant is defined as a pathogenic one. Giannikou et al. reported the same mutation of Family F by sequencing the whole exome of AML tissue (32). The c.1831C>T mutation carried by the Family H is a base substitution in the coding region of the *TSC2* gene, which has been detected by several researchers (31, 33). The mutation of *TSC1* in Family C and J were reported by Dabora et al. (15) and Kwiatkowska et al. (34). In the two families without mutations, the clinical manifestations were not significantly progressed compared with the mutant families. For such patients, early detection and early diagnosis through clinical criteria are important means to control progression. All confirmed and suspected patients need to receive a regular and systematic review and follow-up.

As guided by the 2012 International Tuberous Sclerosis Complex Consensus Conference, genetic diagnosis has now become an independent diagnostic criterion, which helps an early diagnosis of TSC patients with insufficient clinical manifestations, such as infants and children with family history. In addition, for patients with TSC who have been diagnosed through clinical features, a genetic diagnosis can help to determine the type of mutation and provide genetic advice.

## Conclusion

Our study used NGS to identify TSC mutations in 12 Chinese families, including 3 novel *TSC2* variants (c.208dup, c.1874C>G, and c.1852del) and 7 reported variants. In addition, our findings of clinical characteristics of TSC families are consistent with the previous studies. It is strongly recommended that doctors should pay more concentration to typical clinical manifestations of TSC, especially when multiple organs are involved. A comprehensive evaluation is necessary to confirm the diagnosis. At the same time, genetic diagnosis is an important means for those patients whose characteristics do not meet the clinical criteria of TSC. Our results expand the genetic range of TSC and contribute to a more accurate diagnosis and detailed counseling.

## Data Availability Statement

The datasets presented in this article are not readily available because of privacy restrictions on Peking Union Medical Hospital's database. Requests to access the datasets should be directed to beijingzhangyushi@126.com.

## Ethics Statement

Written informed consent was obtained from the individuals and minors' legal guardian for the publication of any potentially identifiable images or data included in this article.

## Author Contributions

YZhang designed the study. XW and WW acquired the data. XW, YZhao, and ZW analyzed the data. XW prepared the first draft. YZhang, YZhao, and ZW reviewed critically and contributed to the final revision. All authors read and approved the final manuscript.

## Funding

The research was supported by the National Natural Science Foundation of China (81670611).

## Conflict of Interest

The authors declare that the research was conducted in the absence of any commercial or financial relationships that could be construed as a potential conflict of interest.

## Publisher's Note

All claims expressed in this article are solely those of the authors and do not necessarily represent those of their affiliated organizations, or those of the publisher, the editors and the reviewers. Any product that may be evaluated in this article, or claim that may be made by its manufacturer, is not guaranteed or endorsed by the publisher.
